# New *Fusarium* species from the Kruger National Park, South Africa

**DOI:** 10.3897/mycokeys.34.25974

**Published:** 2018-06-01

**Authors:** Marcelo Sandoval-Denis, Wijnand J. Swart, Pedro W. Crous

**Affiliations:** 1 Westerdijk Fungal Biodiversity Institute, Uppsalalaan 8, 3584 CT, Utrecht, The Netherlands; 2 Faculty of Natural and Agricultural Sciences, Department of Plant Sciences, University of the Free State, P.O. Box 339, Bloemfontein 9300, South Africa

**Keywords:** Natural parks, phylogeny, fungi, multigene, morphology, diversity

## Abstract

Three new *Fusarium* species, *F. convolutans*, *F. fredkrugeri*, and *F. transvaalense* (Ascomycota, Hypocreales, Nectriaceae) are described from soils collected in a catena landscape on a research supersite in the Kruger National Park, South Africa. The new taxa, isolated from the rhizosphere of three African herbaceous plants, *Kyphocarpa angustifolia*, *Melhania acuminata*, and *Sida cordifolia*, are described and illustrated by means of morphological and multilocus molecular analyses based on sequences from five DNA loci (CAL, EF-1 α, RPB1, RPB2 and TUB). According to phylogenetic inference based on Maximum-likelihood and Bayesian approaches, the newly discovered species are distributed in the *Fusarium buharicum*, *F. fujikuroi*, and *F. sambucinum* species complexes.

## Introduction

Fungi are common colonisers of the plant rhizobiome and endosphere, where they play a key role in modulating the interactions between plant roots and soil (Zachow et al. 2009; Visioli et al. 2014). The direct and indirect interaction between fungal growth in the rhizosphere and its effect on plant growth and health is well documented (Havlicek and Mitchell 2014; Hargreaves et al. 2015; Lareen et al. 2016). Such effects include either a positive feedback by producing plant growth promoting factors, solubilising and stimulating nutrient uptake by plant roots or by inhibiting the growth of concomitant pathogenic organisms (Schippers et al. 1987; Mommer et al. 2016). Conversely, deleterious effects have also been observed, either related to the presence of pathogenic fungal species or caused by fungal-induced modifications of plant root functions, impeding root growth or negatively altering nutrient availability (Schippers et al. 1987; Mommer et al. 2016). Likewise, plants can select and harbour a particular fungal community on its roots via root exudates (Lareen et al. 2016; Sasse et al. 2018), while abiotic influences including water availability, climate and season, soil type, grazers and other animals, orchestrate the development of a unique fungal diversity (Philippot et al. 2013; Havlicek and Mitchell 2014; Hargreaves et al. 2015; Lareen et al. 2016).

The genus *Fusarium* Link (Hypocreales, Nectriaceae) includes a vast number of species, commonly recovered from a variety of substrates including soil, air, water and decaying plant materials; being also able to colonise living tissues of plants and animals, including humans; acting as endophytes, secondary invaders or becoming devastating plant pathogens (Nelson et al. 1994). In addition to their ability to colonise a multiplicity of habitats, *Fusarium* is a cosmopolitan genus, present in almost any ecosystem in the world, including human-made settings such as air and dust in the indoor environment or even in hospitals (Perlroth et al. 2007; Aydogdu and Asan 2008; Pinheiro et al. 2011).

Being common inhabitants of plant root ecosystems, fusaria and, particularly *Fusarium graminearum* Schwabe, *F. proliferatum* (Matsush.) Nirenberg ex Gerlach & Nirenberg, *F. verticillioides* (Sacc.) Nirenberg (Syn. *F. moniliforme* J. Sheld.), *F. oxysporum* Schltdl., as well as species recently segregated from *Fusarium*, including *Neocosmospora phaseoli* (Burkh.) L. Lombard & Crous (Syn. *Fusarium phaseoli* Burkh.) and *N. virguliforme* (O’Donnell & T. Aoki) L. Lombard & Crous (Syn. *F. virguliforme* O’Donnell & T. Aoki), have been regularly studied for their interactions with the rhizobiome, motivated mainly by the importance of these organisms as soil-borne plant pathogens and the need to develop effective control mechanisms (Larkin et al. 1993; Hassan Dar et al. 1997; Pal et al. 2001; Fravel et al. 2003; Idris et al. 2006; Díaz Arias et al. 2013). Similarly, abundant data is available regarding the ecology and distribution of plant-associated fusaria, particularly related to pathogenic species or commonly isolated endophytes (Leslie and Summerell 2006). Little attention has however been given to the occurrence of non-pathogenic fungal species, including *Fusarium* spp. in root microbial communities (Zakaria and Ning 2013; Jumpponen et al. 2017; LeBlanc et al. 2017), while comprehensive DNA sequence-based surveys have been directed mostly to the study of highly relevant and abundant rhizosphere fungal genera such as *Trichoderma* Pers., *Verticillium* Nees or mycorrhizal fungi (Zachow et al. 2009; Bent et al. 2011; Ruano-Rosa et al. 2016; Saravanakumar et al. 2016).

The Kruger National Park (KNP) in South Africa is one of the largest natural reserves in Africa, encompassing a number of non-manipulated landscapes, with almost no human alteration (Carruthers 2017). Recently, four research “supersites” have been identified and established in KNP, each of these supersites representing unique geological, ecological and climatic features of the park (Smit et al. 2013). A multidisciplinary study was conducted in KNP aimed to determine functioning and interaction between abiotic and biotic components, as well as soil properties, hydrology and other processes that determine the structure, biodiversity and heterogeneity of a catena or hill slope ecosystem on one of these “supersites”, located deep inside the KNP (data not published). In order to assess the microbial soil population and community dynamics, mainly focused on bacteria, several rhizosphere samples were obtained from diverse African plants on one of these exceptional protected savannah landscapes. From these collections, interesting fusaria were isolated from the root ecosystem of three native African herbaceous plants i.e. *Kyphocarpa angustifolia* (Moq.) Lopr. (Amaranthaceae), *Melhania acuminata* Mast. (Malvaceae) and *Sida cordifolia* Linn. (Malvaceae). According to their unique morphological traits and clear phylogenetic delimitations, these isolates are described here as three new *Fusarium* species.

## Methods

### Study site and sampling

**Figure 1.**
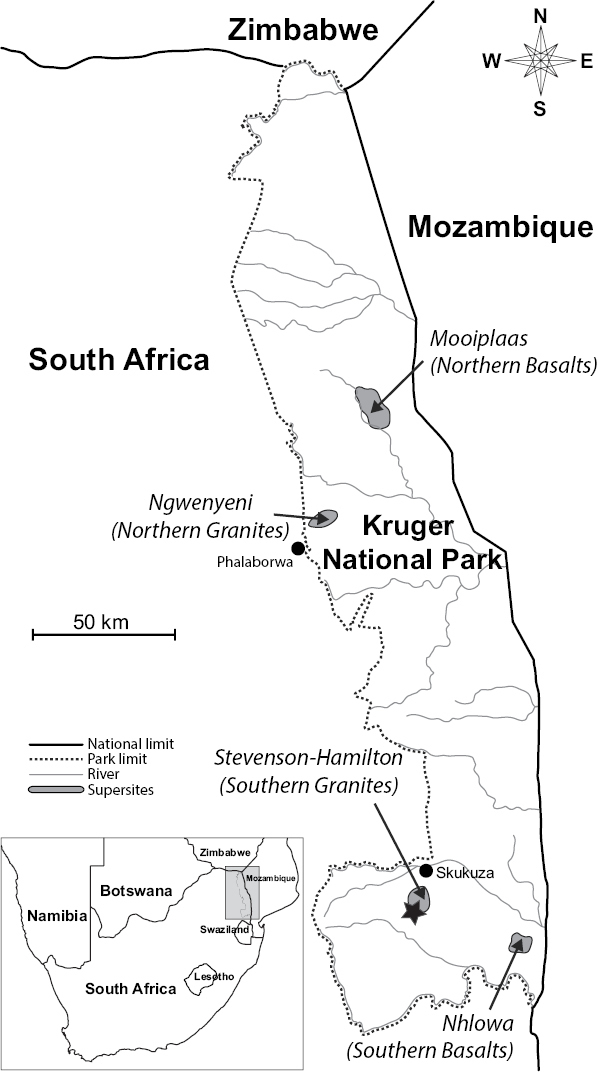
Map of the Kruger National Park (KNP) in South Africa. The arrows indicate the location of the four research “supersites” (adapted from Smit et al. 2013). Sampling site is indicated with a black star. The inset shows the location of the KNP within South Africa, indicated by a grey box.

During March 2015, rhizosphere soil from three herbaceous plants was collected in the Southern Granites “supersite” catena (Stevenson-Hamilton supersite) in the KNP, between 25°06'28.6S, 31°34'41.9E and 25°06'25.7S, 31°34'33.7E (Fig. 1). A catena consists of different soil types observed from a crest to a valley bottom with a wetland or drainage exhibiting different water retention capabilities due to the slope or aspect (topography) and the depth of underlying geological rocks (Brown et al. 2004, Van Zijl and Le Roux 2014). The main characteristics of the Stevenson-Hamilton supersite are described in detail by Smit et al. (2013). Briefly, in this site, a single catena landscape covers approximately 1 km from top to bottom and consists of a hill slope, a sodic site (or grazing lawn), a riparian and floodplain area and a dry drainage line. Three species of plants were selected for sampling occurring at the two extremes of the catena. Two of these species (*Kyphocarpa angustifolia* and *Sida cordifolia*) occurred at both top and bottom sites while *Melhania acuminata* only occurred at the top site. The soil (100 mm depth) at the top of the slope is Clovelly with a high percentage of sand (90%) and a low cation exchange capacity (CEC) (mean sodium concentration of 1062 mg/kg) and pH (mean 5.85). The soil at the bottom of the slope is of the Sterkspruit type, with higher clay content thus higher CEC (mean sodium concentration of 3802 mg/kg) and higher pH (mean 6.4). Rhizosphere soil of 10 plants of the same species occurring at each top or bottom site was sampled using a core soil sampler. A total of 50 samples consisting of ca. 200 g of soil from the roots of each plant were taken, deposited in zip-lock plastic bags and kept on ice in a cool bag at approximately 5 °C until analysed in the laboratory.

### Isolation of *Fusarium* strains

Soil samples were mixed thoroughly and sieved to remove large elements. Fine soil particles were uniformly spread and distributed over the surface of pentachloronitrobenzene agar (PCNB; also known as the Nash-Snyder medium, recipe in Leslie and Summerell 2006) supplemented with streptomycin (0.3 g/l) and neomycin sulphate (0.12 g/l) and malt-extract agar (MEA; recipes on Crous et al. 2009) on 9 mm Petri dishes and incubated at 24 °C for 10 d under a natural day/night photoperiod. Each soil sample was processed in duplicate. Fungal growth was evaluated daily and growing colonies were transferred to fresh Potato Dextrose Agar (PDA; recipe in Crous et al. 2009). Colonies were evaluated for their macro- and microscopic characteristics and a total of 19 fungal cultures showing features typical of *Fusarium* were subjected to single spore isolation as described previously (Sandoval-Denis et al. 2018). Single spore isolates were finally transferred and maintained in Oatmeal Agar plates and slants (OA; recipe in Crous et al. 2009). Fungal strains isolated in this study were deposited in the collection of the Westerdijk Fungal Biodiversity Institute (CBS; Utrecht, the Netherlands), the working collection of Pedro W. Crous (CPC), held at CBS (Table 1); and voucher specimens were deposited in The South African National Collection of Fungi (NCF) (Mycology Unit, Biosystematics Division, Plant Protection Institute, Agricultural Research Council, Pretoria, South Africa).

**Table 1.** Origin, strain and GenBank/ENA accession number of strains and DNA sequences included in this study.

Species name	Strain^†‡^	Country	Host	Sequence accession number^§^				
				*CAL*	*EF-1*α	*RPB1*	*RPB2*	*TUB*
*Fusarium agapanthi*	NRRL 54463^T^	Australia	*Agapanthus* sp.	KU900611	KU900630	KU900620	KU900625	KU900635
*Fusarium ananatum*	CBS 118516^T^	South Africa	*Ananas comosus* fruit	**LT996175**	**LT996091**	**LT996188**	**LT996137**	**LT996112**
*Fusarium andiyazi*	CBS 119857^T^ = NRRL 31727	South Africa	*Sorghum bicolor* soil debris	**LT996176**	**LT996092**	**LT996189**	**LT996138**	**LT996113**
*Fusarium anthophilum*	CBS 737.97 = NRRL 13602	Germany	*Hippeastrum* sp.	**LT996177**	**LT996093**	**LT996190**	**LT996139**	**LT996114**
*Fusarium armeniacum*	NRRL 6227	USA	Fescue hay			JX171446	JX171560	
*Fusarium asiaticum*	CBS 110257 = NRRL 13818	Japan	Barley			JX171459	JX171573	
*Fusarium bactridioides*	NRRL 20476	USA	*Cronartium conigenum*	AF158343	AF160290	Not public	Not public	U34434
*Fusarium begoniae*	CBS 403.97^T^ = NRRL 25300	Germany	*Begonia elatior* hybrid	AF158346	AF160293	**LT996191**	**LT996140**	U61543
*Fusarium buharicum*	CBS 178.35 = NRRL 25488	USSR	*Gossypium* rotting stem base		KX302912	KX302920	KX302928	
	CBS 796.70 = NRRL 13371	Iran	*Hibiscus cannabinus* stalk			JX171449	JX171563	
*Fusarium bulbicola*	CBS 220.76^T^ = NRRL 13618	Germany	*Nerine bowdenii*	KF466327	KF466415	KF466394	KF466404	KF466437
*Fusarium brachygibbosum*	NRRL 13829	Japan	River sediments			JX171460	JX171574	
*Fusarium circinatum*	CBS 405.97^T^ = NRRL 25331	USA	*Pinus radiata*	KM231393	KM231943	JX171510	HM068354	KM232080
*Fusarium coicis*	NRRL 66233^T^	Australia	*Coix gasteenii*	**LT996178**	KP083251	KP083269	KP083274	**LT996115**
*Fusarium concentricum*	CBS 450.97^T^ = NRRL 25181	Costa Rica	*Musa sapientum* fruit	AF158335	AF160282	**LT996192**	JF741086	U61548
*Fusarium continuum*	F201128	China	*Zanthoxylum bungeanum* stem		KM236720	KM520389	KM236780	
*Fusarium convolutans*	CBS 144207^T^ = CPC 33733	South Africa	*Kyphocarpa angustifolia* rhizophere		**LT996094**	**LT996193**	**LT996141**	
	CBS 144208 = CPC 33732	South Africa	*Kyphocarpa angustifolia* rhizophere		**LT996095**	**LT996194**	**LT996142**	
*Fusarium culmorum*	CBS 417.86 = NRRL 25475	Denmark	Moldy barley kernel			JX171515	JX171628	
*Fusarium denticulatum*	CBS 735.97 = NRRL 25302	USA	*Ipomoea batatas*	AF158322	AF160269	**LT996195**	**LT996143**	U61550
*Fusarium dlaminii*	CBS 119860^T^ = NRRL 13164	South Africa	Soil debris in cornfield	AF158330	AF160277	KU171681681	KU171701	U34430
*Fusarium fracticaudum*	CBS 137234^PT^	Colombia	*Pinus maximonoii* stem	**LT996179**	KJ541059	**LT996196**	**LT996144**	KJ541051
*Fusarium fractiflexum*	NRRL 28852^T^	Japan	*Cymbidium* sp.	AF158341	AF160288	Not public	LT575064	AF160315
*Fusarium fredkrugeri*	NRRL 26152	Niger	Unknown		AF160306			AF160321
	CBS 144209^T^ = CPC 33747	South Africa	*Melhania acuminata* rhizophere	**LT996181**	**LT996097**	**LT996199**	**LT996147**	**LT996117**
	CBS 144210 = NRRL 26061	Madagascar	*Striga hermonthica*	AF158356	AF160303	**LT996197**	**LT996145**	AF160319
	CBS 144495 = CPC 33746	South Africa	*Melhania acuminata* rhizophere	**LT996180**	**LT996096**	**LT996198**	**LT996146**	**LT996116**
*Fusarium fujikuroi*	NRRL 13566	China	*Oryza sativa*	AF158332	AF160279	JX171456	JX171570	U34415
*Fusarium globosum*	CBS 428.97^T^ = NRRL 26131	South Africa	*Zea mays*	KF466329	KF466417	KF466396	KF466406	KF466439
*Fusarium goolgardi*	NRRL 66250^T^ = RBG 5411	Australia	*Xanthorrhoea glauca*			KP083270	KP083280	
*Fusarium graminearum*	CBS 123657 = NRRL 31084	USA	Corn			JX171531	JX171644	
*Fusarium konzum*	CBS 119849^T^	USA	*Sorghastrum nuttans*	**LT996182**	**LT996098**	**LT996200**	**LT996148**	**LT996118**
*Fusarium kyushuense*	NRRL 25349	Japan	*Triticum aestivum*				GQ915492	
*Fusarium lactis*	CBS 411.97^NT^ = NRRL 25200	USA	*Ficus carica*	AF158325	AF160272	**LT996201**	**LT996149**	U61551
*Fusarium langsethiae*	NRRL 54940	Norway	Oats			JX171550	JX171662	
*Fusarium lateritium*	NRRL 13622	USA	*Ulmus* sp.		AY707173	JX171457	JX171571	
*Fusarium longipes*	NRRL 13368	Australia	Soil			JX171448	JX171562	
*Fusarium mangiferae*	NRRL 25226	Israel	*Mangifera indica*	AF158334	AF160281	JX171509	HM068353	U61561
*Fusarium mexicanum*	NRRL 47473	Mexico	*Mangifera indica* inflorescence	GU737389	GU737416	Not public	Not public	GU737308
*Fusarium napiforme*	CBS 748.97^T^ = NRRL 13604	Namibia	*Pennisetum typhoides*	AF158319	AF160266	HM347136	EF470117	U34428
*Fusarium nygamai*	CBS 749.97^T^ = NRRL 13448	Australia	*Sorghum bicolor* necrotic root	AF158326	AF160273	**LT996202**	EF470114	U34426
*Fusarium oxysporum*	CBS 716.74 = NRRL 20433	Germany	*Vicia faba* vascular bundle	AF158366	AF008479	JX171469	JX171583	U34435
	CBS 744.97 = NRRL 22902	USA	*Pseudotsuga menziesii*	AF158365	AF160312	**LT996203**	LT575065	U34424
*Fusarium palustre*	NRRL 54056^T^	USA	*Spartina alterniflora*			KT597718	KT597731	
*Fusarium parvisorum*	CBS 137236^T^	Colombia	*Pinus patula* roots	**LT996183**	KJ541060		**LT996150**	KJ541055
*Fusarium phyllophilum*	CBS 216.76^T^ = NRRL 13617	Italy	*Dracaena deremensis* leaf	KF466333	KF466421	KF466399	KF466410	KF466443
*Fusarium poae*	NRRL 13714	Unknown	Unknown			JX171458	JX171572	
*Fusarium proliferatum*	CBS 217.76 = NRRL 22944	Germany	*Cattleya* pseudobulb, hybrid	AF158333	AF160280	JX171504	HM068352	U34416
*Fusarium pseudocircinatum*	CBS 449.97^T^ = NRRL 22946	Ghana	*Solanum* sp.	AF158324	AF160271	**LT996204**	**LT996151**	U34427
*Fusarium pseudograminearum*	CBS 109956^T^ = NRRL 28062	Australia	*Hordeum vulgare* crowns			JX171524	JX171637	
*Fusarium pseudonygamai*	CBS 417.97^T^ = NRRL 13592	Nigeria	*Pennisetum typhoides*	AF158316	AF160263	**LT996205**	**LT996152**	U34421
*Fusarium ramigenum*	CBS 418.98^T^ = NRRL 25208	USA	*Ficus carica*	KF466335	KF466423	KF466401	KF466412	KF466445
*Fusarium sacchari*	CBS 223.76 = NRRL 13999	India	*Saccharum officinarum*	AF158331	AF160278	JX171466	JX171580	U34414
*Fusarium sambucinum*	NRRL 22187 = NRRL 20727	England	*Solanum* sp.			JX171493	JX171606	
*Fusarium sarcochroum*	CBS 745.79 = NRRL 20472	Switzerland	*Viscum album*			JX171472	JX171586	
*Fusarium sibiricum*	NRRL 53430^T^	Russia	*Avena sativa*				HQ154472	
*Fusarium sororula*	CBS 137242^T^	Colombia	*Pinus patula* stems	**LT996184**	KJ541067	**LT996206**	**LT996153**	KJ541057
*Fusarium* sp.	NRRL 66179	USA	*Hibiscus moscheutos*		KX302913	KX302921	KX302929	
	NRRL 66180	USA	*Hibiscus moscheutos*		KX302914	KX302922	KX302930	
	NRRL 66181	USA	*Hibiscus moscheutos*		KX302915	KX302923	KX302931	
	NRRL 66182	USA	*Hibiscus moscheutos*		KX302916	KX302924	KX302932	
	NRRL 66183	USA	*Hibiscus moscheutos*		KX302917	KX302925	KX302933	
	NRRL 66184	USA	*Hibiscus moscheutos*		KX302918	KX302926	KX302934	
	CBS 201.63 = NRRL 36351	Portugal	*Arachis hypogaea* stored nut				GQ915484	
*Fusarium sporotrichioides*	NRRL 3299	USA	Corn			JX171444	HQ154454	
*Fusarium sterilihyphosum*	NRRL 25623	South Africa	Mango	AF158353	AF160300	Not public	Not public	AF160316
*Fusarium stilboides*	NRRL 20429	Nyasaland	Coffee bark			JX171468	JX171582	
*Fusarium subglutinans*	CBS 747.97 = NRRL 22016	USA	Corn	AF158342	AF160289	JX171486	JX171599	U34417
*Fusarium sublunatum*	CBS 190.34 = NRRL 20897	Unknown	Unknown		KX302919	KX302927	KX302935	
	CBS 189.34^T^ = NRRL 13384	Costa Rica	Soil of banana plantation			JX171451	JX171565	
*Fusarium succisae*	CBS 219.76 = NRRL 13613	Germany	*Succisa pratensis* flower	AF158344	AF160291	**LT996207**	**LT996154**	U34419
*Fusarium sudanense*	CBS 454.97^T^ = NRRL 25451	Sudan	*Striga hermonthica*	**LT996185**	KU711697	**LT996208**	**LT996155**	KU603909
*Fusarium temperatum*	NRRL 25622 = NRRL 26616	South Africa	*Zea mays*	AF158354	AF160301	Not public	Not public	AF160317
*Fusarium terricola*	CBS 483.94^T^	Australia	Soil	KU603951	KU711698	**LT996209**	**LT996156**	KU603908
*Fusarium thapsinum*	CBS 733.97 = NRRL 22045	South Africa	*Sorghum bicolor*	**LT996186**	AF160270	JX171487	JX171600	U34418
*Fusarium tjaetaba*	NRRL 66243^T^	Australia	*Sorghum interjectum*	**LT996187**	KP083263	KP083267	KP083275	**LT996119**
*Fusarium torreyae*	NRRL 54149	USA	*Torreya* sp.		HM068337	JX171548	HM068359	
*Fusarium transvaalense*	CBS 144211^T^ = CPC 30923	South Africa	*Sida cordifolia* rhizosphere		**LT996099**	**LT996210**	**LT996157**	**LT996120**
	CBS 144212 = CPC 30929	South Africa	*Melhania acuminata* rhizophere		**LT996100**	**LT996211**	**LT996158**	**LT996121**
	CBS 144213 = CPC 33751	South Africa	*Melhania acuminata* rhizophere				**LT996159**	**LT996122**
	CBS 144214 = CPC 30946	South Africa	*Sida cordifolia* rhizosphere		**LT996101**	**LT996212**	**LT996160**	**LT996123**
	CBS 144215 = CPC 33723	South Africa	*Sida cordifolia* rhizosphere		**LT996102**		**LT996161**	**LT996124**
	CBS 144216 = CPC 30918	South Africa	*Sida cordifolia* rhizosphere		**LT996103**	**LT996213**	**LT996162**	**LT996125**
	CBS 144217 = CPC 30919	South Africa	*Sida cordifolia* rhizosphere		**LT996104**	**LT996214**	**LT996163**	**LT996126**
	CBS 144218 = CPC 30922	South Africa	*Sida cordifolia* rhizosphere		**LT996105**	**LT996215**	**LT996164**	**LT996127**
	CBS 144219 = CPC 30926	South Africa	*Sida cordifolia* rhizosphere		**LT996106**	**LT996216**	**LT996165**	**LT996128**
	CBS 144220 = CPC 30927	South Africa	*Sida cordifolia* rhizosphere		**LT996107**	**LT996217**	**LT996166**	**LT996129**
	CBS 144221 = CPC 33740	South Africa	*Kyphocarpa angustifolia* rhizophere				**LT996167**	**LT996130**
	CBS 144222 = CPC 30939	South Africa	*Kyphocarpa angustifolia* rhizophere		**LT996108**	**LT996218**	**LT996168**	**LT996131**
	CBS 144223 = CPC 30941	South Africa	*Kyphocarpa angustifolia* rhizophere		**LT996109**		**LT996169**	**LT996132**
	CBS 144224 = CPC 30928	South Africa	*Melhania acuminata* rhizophere		**LT996110**	**LT996219**	**LT996170**	**LT996133**
	CBS 144496 = CPC 33750	South Africa	*Melhania acuminata* rhizophere				**LT996171**	**LT996134**
	NRRL 31008	Australia	Soil			JX171529	JX171642	
*Fusarium tupiense*	NRRL 53984	Brazil	*Mangifera indica*	GU737377	GU737404	Not public	Not public	GU737296
*Fusarium udum*	CBS 178.32 = NRRL 22949	Germany	*Lactarius pubescens*	AF158328	AF160275	**LT996220**	**LT996172**	U34433
*Fusarium venenatum*	CBS 458.93^T^	Austria	Winter wheat halm base				KM232382	
*Fusarium verticillioides*	CBS 734.97 = NRRL 22172	Germany	*Zea mays*	AF158315	AF160262	**LT996221**	EF470122	U34413
*Fusarium xanthoxyli*	F201114	China	*Zanthoxylum bungeanum*		KM236706	KM520380	KM236766	
*Fusarium xylarioides*	CBS 258.52 = NRRL 25486	Ivory Coast	*Coffea* sp. trunk		AY707136	JX171517	HM068355	AY707118

^†^CBS: Westerdijk Fungal Biodiversity Institute. CPC: Collection of Pedro W. Crous, held at CBS. F: College of Forestry, Northwest A&F University, Taicheng Road, Yangling, Shaanxi China. NRRL: Agricultural Research Service, Peoria, IL, USA.

^‡ IT^: ex-isotype culture. ^PT^: ex-paratype culture. ^T^: ex-type culture. ^NT^: ex-neotyype culture.

^§^*CAL*: Calmodulin. *EF-1α*: Translation elongation factor 1-alpha. *RPB1*: RNA polymerase largest subunit. *RPB2*: RNA polymerase second largest subunit. *TUB*: Tubulin. New sequences are shown in **bold**. Sequences marked as “Not public” were obtained from Kerry O’Donnell’s alignment datasets.

### Morphological characterisation

*Fusarium* isolates were characterised morphologically according to procedures described elsewhere (Aoki et al. 2013; Leslie and Summerell 2006, Sandoval-Denis et al. 2018). Colonial growth rates and production of diffusible pigments were evaluated on PDA, colony features were also recorded on corn-meal agar (CMA; recipe in Crous et al. 2009) and OA. Colour notations followed those of Rayner (1970). For the study of micro-morphological features, cultures were grown for 7–10 d at 24 °C, using a 12 h light/dark cycle with near UV and white fluorescent light. Aerial and sporodochial conidiophores and conidia and formation of chlamydospores were evaluated on Synthetic Nutrient-poor Agar (SNA; Nirenberg 1976) and on Carnation Leaf Agar (CLA; Fisher et al. 1982). Measurements and photomicrographs were recorded from a minimum of 30 elements for each structure, using sterile water as mounting medium and a Nikon Eclipse 80i microscope with Differential Interference Contrast (DIC) optics and a Nikon AZ100 dissecting microscope, both equipped with a Nikon DS-Ri2 high definition colour digital camera and the Nikon software NIS-elements D software v. 4.30.

### DNA isolation, amplification and sequencing

Isolates were grown for 7 d on MEA at 24 °C using the photoperiod described above. Fresh mycelium was scraped from the colony surface and subjected to total DNA extraction using the Wizard® Genomic DNA purification Kit (Promega Corporation, Madison, WI, USA), according to the manufacturer’s instructions. Fragments of five DNA loci were amplified using primers and PCR conditions described by O’Donnell et al. (2009) for calmodulin (*CAL*), O’Donnell et al. (2010) for the RNA polymerase largest subunit (*RPB1*) and second largest subunit (*RPB2*), O’Donnell et al. (1998) for the translation elongation factor 1-alpha (*EF-1α*) and Woudenberg et al. (2009) for beta-tubulin (*TUB*). Sequencing was made in both strand directions using the same primer pairs as for PCR amplification on an Applied Biosystems, Hitachi 3730xl DNA analyser (Applied Biosystems Inc., Foster City, California, USA). Consensus sequences were assembled using Seqman Pro v. 10.0.1 (DNASTAR, Madison, WI, USA). All DNA sequences generated in this study were lodged in GenBank and the European Nucleotide Archive (ENA) (Table 1).

### Molecular identification and phylogenetic analyses

A first analysis was based on pairwise alignments and blastn searches on the *Fusarium* MLST (http://www.westerdijkinstitute.nl/fusarium/) and NCBI (https://blast.ncbi.nlm.nih.gov/Blast.cgi) databases, respectively, using *EF-1α* and *RPB2* sequences in order to resolve the position of the KNP isolates amongst the different species complexes recognised in *Fusarium* (O’Donnell et al. 2013). Sequences from individual loci were aligned using MAFFT (Katoh and Standley 2013), on the web server of the European Bioinformatics Institute (EMBL–EBI; http://www.ebi.ac.uk/Tools/msa/mafft/) (Li et al. 2015).

Phylogenetic analyses were based on Maximum-likelihood (ML) and Bayesian (B) analyses, both algorithms run on the CIPRES Science Gateway portal (Miller et al. 2012). Evolutionary models were calculated using MrModelTest v. 2.3 using the Akaike information criterion (Nylander 2004; Posada and Crandall 1998). For ML, RAxML-HPC2 v. 8.2.10 on XSEDE was used (Stamatakis 2014), clade stability was tested with a bootstrap analysis (BS) using the rapid bootstrapping algorithm with default parameters. The B analyses were run using MrBayes v. 3.2.6 on XSEDE (Ronquist and Huelsenbeck 2003) using four incrementally heated MCMC chains for 5M generations, with the stop-rule option on and sampling every 1000 trees. After convergence of the runs (average standard deviation of split frequencies below 0.01) the first 25% of samples were discarded as the burn-in fraction and 50% consensus trees and posterior probabilities (PP) were calculated from the remaining trees.

Phylogenies were first made individually for each locus dataset and visually compared for topological incongruence amongst statistically supported nodes (ML-BS ≥ 70% and B-PP ≥ 0.95) (Mason-Gamer and Kellogg 1996, Wiens 1998), before being concatenated for multi-locus analyses using different locus combinations according to strains and DNA sequences currently available in public databases, in addition to previously published phylogenies (O’Donnell et al. 2000, 2013; Herron et al. 2015; Lupien et al. 2017; Moussa et al. 2017, Sandoval-Denis et al. 2018). A further 232 sequences representing 72 taxa were retrieved from GenBank and included in the phylogenetic analyses, while an additional 58 DNA sequences were obtained from 24 fungal strains requested from the CBS and NRRL (Agricultural Research Service, Peoria, IL, USA) culture collections (Table 1). All alignments and trees generated in this study were uploaded to TreeBASE (https://treebase.org).

## Results

### Phylogenetic analyses

Pairwise DNA alignments and BLAST searches using *EF-1α* and *RPB2* sequences showed that the 19 isolates from KNP belonged to three different species complexes of the genus *Fusarium* i.e. the *F. buharicum* Jacz. ex Babajan & Teterevn.-Babajan species complex (FBSC; two isolates), the *F. fujikuroi* Nirenberg species complex (FFSC; two isolates) and the *F. sambucinum* Fuckel species complex (FSAMSC; 15 isolates). According to these results, sequences of related taxa and lineages were retrieved from GenBank and incorporated into individual phylogenetic analyses for each species complex.

Multi-locus analyses were carried out in order to further delimit the KNP *Fusarium* isolates amongst the known diversity in their respective species complexes. With the exception of the FFSC, the topologies observed from ML and B analyses of single and multi-locus datasets were highly congruent, with only minor differences affecting unsupported nodes on the trees (all trees available in TreeBASE). The characteristics of the different alignments and tree statistics for all the species complexes are shown in Table 2.

**Table 2.** Characteristics of the different datasets and statistics of phylogenetic analyses used in this study.

Analysis^†^	Locus^‡^	Number of Sites^§^				Evolutionary model^|^	Number of trees sampled in B	Maximum-likelihood statistics	
		Total	Conserved	Phylogenetically informative	B unique patterns			Best tree optimised likelihood	Tree length
*Fusarium buharicum* SC	*EF-1α*	495	300	119	198	GTR+G	414	-11313.23702	0.598675
	*RPB1*	930	682	203	211	SYM+G			
	*RPB2*	1663	1251	330	310	GTR+I+G			
*Fusarium fujikuroi* SC	*CAL*	545	423	67	167	SYM+G	282	-20603.30043	0.567054
	*EF-1α*	677	428	127	295	GTR+I+G			
	*RPB1*	1534	1219	185	137	SYM+I+G			
	*RPB2*	1551	1211	227	315	GTR+I+G			
	*TUB*	488	351	66	336	SYM+G			
*Fusarium sambucinum* SC	*RPB1*	854	594	201	213	SYM+I+G	241	-9871.793718	0.740271
	*RPB2*	1580	1128	346	396	GTR+G			

^†^ SC: Species complex.

^‡^*CAL*: Calmodulin. *EF-1α*: Translation elongation factor 1-alpha. *RPB1*: RNA polymerase largest subunit. *RPB2*: RNA polymerase second largest subunit. *TUB*: Tubulin.

^§^ B: Bayesian inference.

^|^ G: Gamma distributed rate variation among sites. GTR: Generalised time-reversible. I: Proportion of invariable sites. SYM: Symmetrical model.

**Figure 2.**
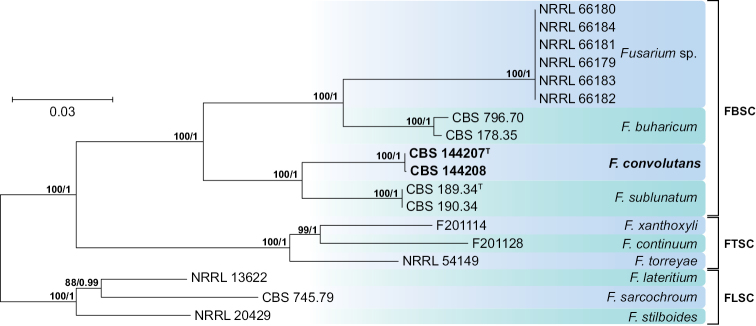
Maximum-likelihood (ML) phylogram obtained from combined *EF-1α*, *RPB1* and *RPB2* sequences of 18 strains belonging to the *Fusarium buharicum* (FBSC), *Fusarium tricinctum* (FTSC) and *Fusarium lateritium* (FLSC) species complexes. Numbers on the nodes are ML bootstrap values above 70% and Bayesian posterior probability values above 0.95. Branch lengths are proportional to distance. Ex-type strains are indicated with ^T^. Strains corresponding to new species described here are shown in **bold**.

The analysis of the FBSC included sequences of *EF-1α, RPB1* and *RPB2* loci from 18 isolates representing 10 taxa, including members of the *Fusarium torreyae* T. Aoki, J.A. Sm., L.L. Mount, Geiser & O’Donnell species complex (FTYSC) and *Fusarium lateritium* Nees species complex (FLSC) as outgroup (Fig. 2). The four ingroup taxa resolved with high statistical support. Two KNP isolates from *K. angustifolia* obtained from the bottom site of the catena (CBS 144207 and 144208) clustered in a sister relationship with the clade representing *Fusarium sublunatum* Reinking, but were genetically clearly delimited.

**Figure 3.**
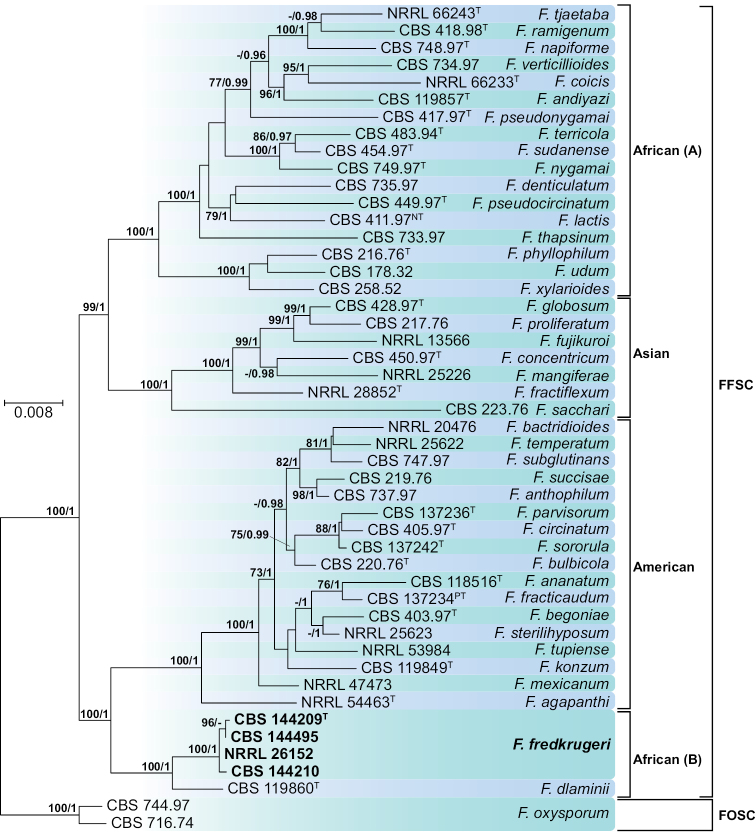
Maximum-likelihood (ML) phylogram obtained from combined *CAL*, *EF-1α*, *RPB1*, *RPB2* and *TUB* sequences of 48 strains belonging to the *Fusarium fujikuroi* (FFSC) and *Fusarium oxysporum* (FOSC) species complexes. Numbers on the nodes are ML bootstrap values above 70% and Bayesian posterior probability values above 0.95. Branch lengths are proportional to distance. Ex-type, ex-neotype and ex-paratype strains are indicated with ^T, NT^ and ^PT^, respectively. Strains corresponding to new species described here are shown in **bold**.

The phylogeny of the FFSC included sequences of *CAL, EF-1α, RPB1, RPB2* and *TUB* loci from 48 strains and 44 taxa, including two outgroups (*F. oxysporum* CBS 716.74 and 744.97) (Fig. 3). The phylogeny showed a clear delimitation between the biogeographic clades recognised in this species complex (African, American and Asian clades *sensu* O’Donnell et al. 1998). Both American and Asian clades where shown as monophyletic with high ML-BS and B-PP support; in contrast, the African clade was resolved as polyphyletic, comprising two distinct and highly supported lineages. A terminal, speciose clade (African A) encompassing 17 taxa and a basal clade (African B), close to the American clade which included the ex-type of *Fusarium dlaminii* Marasas, P.E. Nelson & Toussoun (CBS 119860) and a sister terminal clade (ML-BS=100, B-PP=1) comprising two KNP isolates from *M. acuminata* (CBS 144209 and 144495) and two unidentified African *Fusarium* isolates (CBS 144210 and NRRL 26152). From the loci used here, only *TUB* resolved both African clades as sister groups; however, its monophyly was not supported by clade stability measurements (data not shown). Conversely, individual *CAL, EF-1α* and *RPB2* phylogenies resolved African B as basal to the ingroup, while *RPB1* allocated this clade as basal to the American clade. Nonetheless, all the individual phylogenies, in addition to the combined dataset, clearly demonstrated genealogical uniqueness of the terminal clade encompassing KNP isolates.

**Figure 4.**
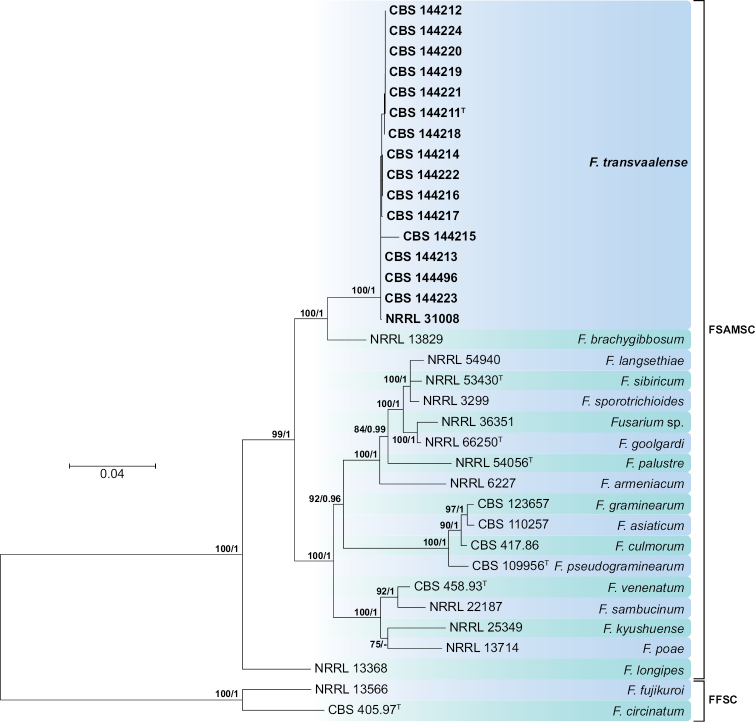
Maximum-likelihood (ML) phylogram obtained from combined *RPB1* and *RPB2* sequences of 35 strains belonging to the *Fusarium sambucinum* (FSAMSC) and *Fusarium fujikuroi* (FFSC) species complexes. Numbers on the nodes are ML bootstrap values above 70% and Bayesian posterior probability values above 0.95. Branch lengths are proportional to distance. Ex-type strains are indicated with ^T^. Strains corresponding to new species described here are shown in **bold**.

The FSAMSC was studied using combined *RPB1* and *RPB2* sequences. The phylogeny included 35 isolates from 20 taxa, including the two outgroups *Fusarium circinatum* Nirenberg & O’Donnell (CBS 405.97) and *Fusarium fujikuroi* Nirenberg (NRRL 13566) (Fig. 4). Fifteen KPN *Fusarium* isolates from the three sampled plant species (three isolates from *K. angustifolia*, four isolates from *M. acuminata* and eight isolates from *S. cordifolia*), all obtained from the top site of the catena, clustered with an unidentified *Fusarium* isolate (NRRL 31008) in a distinct clade (ML-BS=100, B-PP=1), close to *Fusarium brachygibbosum* Padwick (strain NRRL 13829).

The clades including KNP isolates and corresponding to previously undisclosed lineages of Fusarium are described in the taxonomy section as the three novel species, *F. convolutans*, *F. fredkrugeri* and *F. transvaalense*.

### Taxonomy

#### Fusarium convolutans

Taxon classificationFungiHypocrealesNectriaceae

Sandoval-Denis, Crous & W.J. Swart sp. nov.

MB825102

Fig. 5

##### Diagnosis.

Different from *F. circinatum*, *F. pseudocircinatum* O’Donnell & Nirenberg and *F. sterilihyphosum* Britz, Marasas & M.J. Wingf. by the absence of aerial conidia (microconidia) and the presence of chlamydospores. Different from *F. buharicum* Jacz. ex Babajan & Teterevn.-Babajan and *F. sublunatum* by its shorter, less septate and less curved conidia and by the presence of sterile hyphal coils.

##### Type.

South Africa, Kruger National Park, Skukuza, Granite Supersite, 25°06'33.9"S, 31°34'40.9E, from rhizosphere soil of *Kyphocarpa angustifolia*, 23 Mar 2015, W.J. Swart, holotype CBS H-23495, dried culture on OA, ex-holotype strain CBS 144207 = CPC 33733.

##### Description.

Colonies on PDA growing in the dark with an average radial growth rate of 2.1–4.8 mm/d, 4.4–5.8 mm/d and 4.6–6.3 mm/d at 24, 27 and 30 °C, respectively; reaching 11–28 mm diam. in 7 d at 24 °C and a maximum of 23–37 mm diam. in 7 d at 30 °C. Minimum temperature for growth 12 °C, maximum 36 °C, optimal 27–33 °C. Colony surface white to cream coloured, flat and highly irregular in shape, velvety to felty, with scant and short aerial mycelium; colony margins highly irregular to rhizoid, with abundant white to grey submerged mycelium. Reverse white, straw to yellow diffusible pigment produced between 21–33 °C, scarcely produced and turning luteous to orange at 36 °C. Colonies on CMA and OA incubated in the dark reaching 40–48 mm diam. in 7 d at 24 °C. Colony surface white to cream coloured, flat or slightly elevated at the centre, velvety to dusty; aerial mycelium abundant, short and dense, concentrated on the colony centre; margins membranous and regular, buff to honey coloured, without aerial mycelium. Reverse ochreous without diffusible pigments. Sporulation scant from conidiophores formed on the aerial mycelium, sporodochia not formed. *Conidiophores* on the aerial mycelium straight or flexuous, smooth- and thin-walled, simple, mostly reduced to conidiogenous cells borne laterally on hyphae or up to 50 μm tall, bearing terminal single or paired monophialides; *phialides* subulate to subcylindrical, smooth- and thin-walled, 15.5–22 μm long, (3.5–)4–5 μm at the widest point, with inconspicuous periclinal thickening and a short- flared collarette; *conidia* clustering in discrete false heads at the tip of monophialides, lunate to falcate, curved or somewhat straight, tapering gently toward the basal part, robust; apical cell often equal in length or slightly shorter than the adjacent cell, blunt to conical; basal cell papillate to distinctly notched, (1–2–)3-septate, hyaline, thin- and smooth-walled. One-septate conidia: 24 × 4.5 μm; two-septate conidia: 24.5 × 6 μm; three-septate conidia: (25.5–)29–36.5(–38.5) × (4–)5–6.5(–7.5) μm. *Chlamydospores* abundantly formed, globose to subglobose, smooth- and thick-walled, (9.5–)11–13.5(–14) μm diam.; terminal or intercalary in the hyphae or conidia, often borne laterally at the tip of elongated, cylindrical, stalk-like projections, solitary or in small clusters. Sterile, coiled, sometimes branched hyphal projections abundantly formed laterally from the substrate and aerial mycelium.

**Figure 5.**
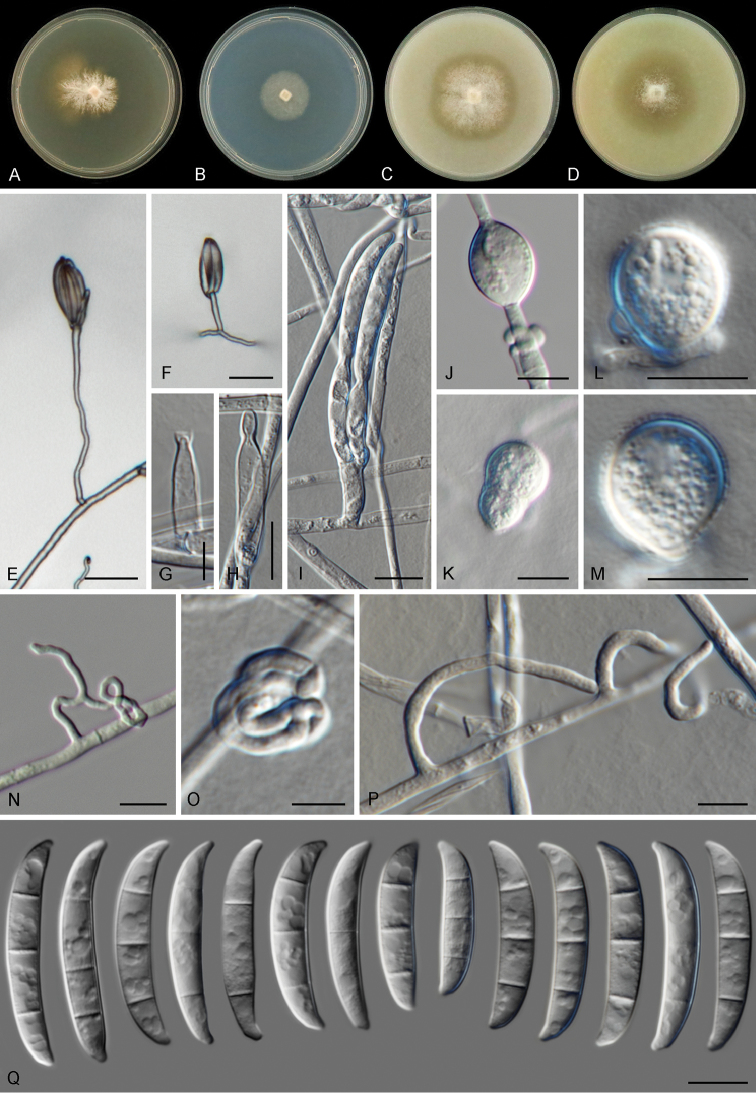
*Fusarium convolutans* sp. nov. **A–D** Colonies on PDA, SNA, OA and CMA, respectively, after 7 d at 24 °C in the dark **E–I** Conidiophores, phialides and conidia **J–M** Chlamydospores **N–P** Sterile hyphal projections **Q** Conidia. Scale bars: 20 μm (**E, F**); 5 μm (**G–I)**; 10 μm (**J–Q**).

##### Distribution.

South Africa.

##### Etymology.

From Latin, “convolutans”, participle of *convolutare*, coiling, in reference to the abundant sterile, coiled lateral hyphal projections.

##### Additional isolate examined.

South Africa, Kruger National Park, Skukuza, Granite Supersite, 25°06'33.9"S, 31°34'40.9E, from rhizosphere soil of *Kyphocarpa angustifolia*, 23 Mar 2015, W.J. Swart, CBS 144208 = CPC 33732.

##### Notes.

The main morphological feature of *F. convolutans*, namely the production of sterile, coiled hyphal projections, grossly resembles other *Fusarium* species producing similar structures i.e. *F. circinatum*, *F. pseudocircinatum* and *F. sterilihyphosum*. The three latter species, however, are genetically unrelated to *F. convolutans*, being allocated in the FFSC; and are also easily differentiable by the characteristics of the aerial conidia (typical *Fusarium* microconidia are absent in the new species) and the lack of chlamydospores (present in the new species) (Leslie and Summerell 2006). *Fusarium convolutans* can be easily differentiated morphologically from their phylogenetically closely related species, *F. buharicum* and *F. sublunatum.* It has relative simple conidiophores and shorter, less septate and markedly less curved conidia (up to 38.5 μm long and 1–3-septate vs. up to 87 and 81 μm long, 0–8-septate in *F. buharicum* and *F. sublunatum*, respectively) (Gerlach and Nirenberg 1982). *Fusarium buharicum* and *F. sublunatum* also lack sterile hyphal coils.

#### Fusarium fredkrugeri

Taxon classificationFungiHypocrealesNectriaceae

Sandoval-Denis, Crous & W.J. Swart sp. nov.

MB825103

Fig. 6

##### Diagnosis.

Differs from *Fusarium dlaminii* Marasas, P.E. Nelson & Toussoun by producing only one type of aerial conidia, shorter sporodochial conidia and the absence of chlamydospores.

##### Type.

South Africa, Kruger National Park, Skukuza, Granite Supersite, 25°06'48.6"S, 31°34'36.5"E, from rhizosphere soil of *Melhania acuminata*, 23 Mar 2015, W.J. Swart, holotype CBS H-23496, dried culture on OA, culture ex-holotype CBS 144209 = CPC 33747.

##### Description.

Colonies on PDA growing in the dark with an average radial growth rate of 4.7–5.8 mm/d and reaching 22–35 mm diam. in 7 d at 24 °C, filling an entire 9 cm Petri dish in 7 d at 27 and 30 °C. Minimum temperature for growth 12 °C, maximum 36 °C, optimal 27–30 °C. Colony surface at first white to cream coloured, later turning bay to chestnut with pale luteous to luteous periphery; flat, felty to cottony with abundant erect- aerial mycelium forming white patches; colony margins regular and filiform with abundant submerged mycelium. Reverse pale luteous, a blood sepia to chestnut coloured diffusible pigment is scarcely produced at 24 °C, pigment production is markedly enhanced at 27–30 °C, becoming greyish-sepia at 33 °C. Colonies on CMA and OA incubated at 24 °C in the dark reaching 65–67 mm diam. or occupying an entire 9 cm Petri dish in 7 d, respectively. Colony surface pale bay coloured, flat, felty to velvety, aerial mycelium scant, forming white to cream patches; margins regular. Reverse pale bay to pale vinaceous. Sporulation abundant from conidiophores formed on the substrate and aerial mycelium and from sporodochia. *Conidiophores* on the aerial mycelium straight or flexuous, erect or prostrate, septate, smooth- and thin-walled, often appearing rough by accumulation of extracellular material, commonly simple or reduced to conidiogenous cells borne laterally on hyphae or up to 200 μm tall and irregularly branched at various levels, branches bearing lateral and terminal monophialides borne mostly single or in pairs; *phialides* subulate, ampulliform, lageniform to subcylindrical, smooth- and thin-walled, (8.5–)9.5–17.5(–24.5) μm long, 2–3(–3.5) μm at the widest point, without periclinal thickening, collarets inconspicuous; *conidia* formed on aerial conidiophores, hyaline, obovoid, ellipsoidal to slightly reniform or allantoid, smooth- and thin-walled, 0-septate, (4.5–)5–8.5(–12.5) × (1.5–)2–3.5(–6) μm, clustering in discrete false heads at the tip of monophialides. *Sporodochia* pale orange to pink coloured, often somewhat translucent, formed abundantly on the surface of carnation leaves and on the agar surface. *Conidiophores* in sporodochia 26–46 μm tall, densely aggregated, irregularly and verticillately branched up to three times, with terminal branches bearing 2–3 monophialides; *sporodochial phialides* doliiform to subcylindrical, (9–)11.5–15.5(–18.5) × (2.5–)3–4(–4.5) μm, smooth- and thin-walled, with periclinal thickening and an inconspicuous apical collarette. *Sporodochial conidia* falcate, tapering toward the basal part, robust, moderately curved and slender; apical cell more or less equally sized than the adjacent cell, blunt to slightly papillate; basal cell papillate to distinctly notched, (1–)3–4-septate, hyaline, thin- and smooth-walled. One-septate conidia: 13–17(–18) × (2.5–)3–4 μm; two-septate conidia: 15 × 4.5 μm; three-septate conidia: (16–)28.5–39(–45) × (3–)4–5(–5.5) μm; four-septate conidia: 39.5–40(–41) × 4.5–5 μm; overall (13–)27.5–39.5(–45) × (3–)3.5–5.5 μm. Chlamydospores absent.

**Figure 6.**
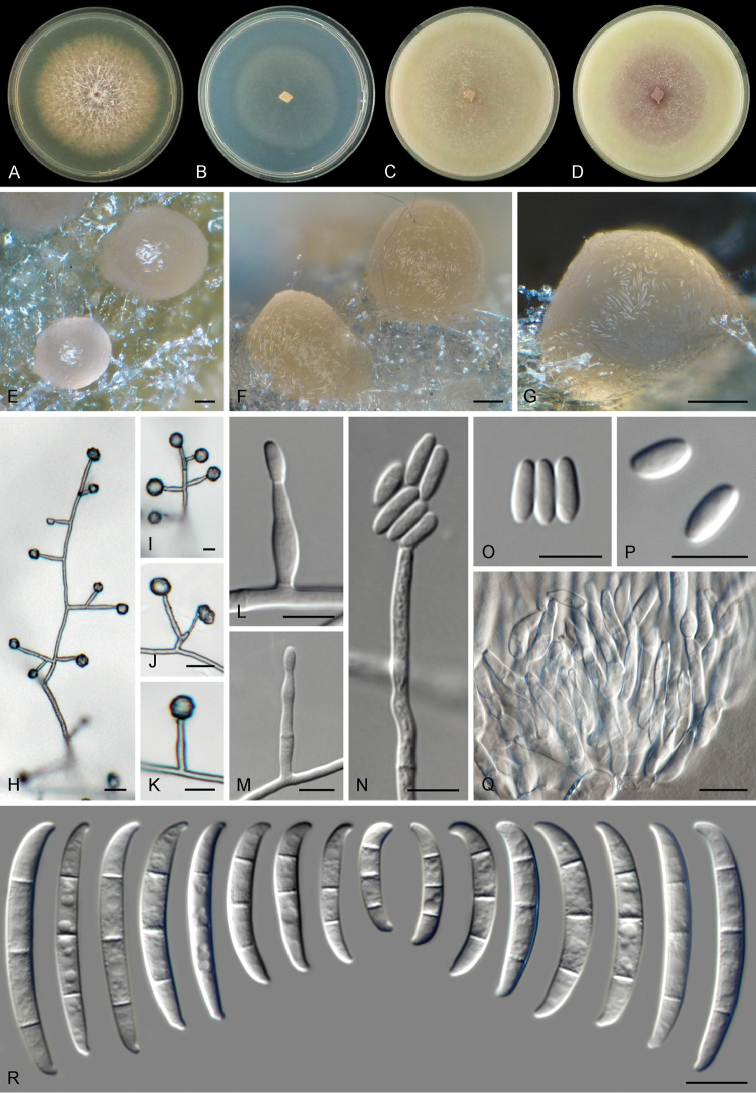
*Fusarium fredkrugeri* sp. nov. **A–D** Colonies on PDA, SNA, OA and CMA, respectively, after 7 d at 24 °C in the dark **E–G** Sporodochia formed on the surface of carnation leaves **H–N** Aerial conidiophores, phialides and conidia **O, P** Aerial conidia **Q** Sporodochial conidiophores and phialides **R** Sporodochial conidia. Scale bars: 100 μm (**E–G)**; 10 μm (**H–R**).

##### Distribution.

Madagascar, Niger and South Africa.

##### Etymology.

In honour and memory of Dr. Frederick J. Kruger, pioneer of forest hydrology, fynbos ecology and invasive species and fundamental for the collections included in this study.

##### Additional isolates examined.

Madagascar, from *Striga hermonthica*, unknown date, A.A. Abbasher, CBS 144210 = NRRL 26061 = BBA 70127. South Africa, Kruger National Park, Skukuza, Granite Supersite,25°06'48.6"S, 31°34'36.5"E, from rhizosphere soil of *Melhania acuminata*, 23 Mar 2015, W.J. Swart, CBS 144495 = CPC 33746.

##### Notes.

This species is genetically closely related to *F. dlaminii*, both species having similar colonial morphology, optimal growth conditions and biogeography. Moreover, both species exhibit relatively short aerial phialides producing conidia in heads, somewhat resembling those produced by *F. oxysporum* rather than most members of the FFSC (Leslie and Summerell 2006; Marasas et al. 1985). However, besides exhibiting much faster growth rates, *F. fredkrugeri* presents clearly distinctive morphological features such as the production of only one type of aerial conidia (vs. two types in *F. dlaminii*: allantoid to fusiform and 0-septate; and napiform 0–1-septate); orange to pink sporodochia, produced on carnation leaves but also abundantly on the agar surface (vs. orange sporodochia, produced only on the surface of carnation leaves in *F. dlaminii*) (Leslie and Summerell 2006). Additionally, *F. fredkrugeri* produces shorter and less septate sporodochial conidia ((1–)3–4-septate and up to 45 μm long in the latter species vs. mostly 5-septate and up to 54 μm long in *F. dlaminii*) while chlamydospores are not produced. The latter feature, coupled with the somewhat more complex conidiophores also clearly differentiates *F. fredkrugeri* from *F. oxysporum*.

#### Fusarium transvaalense

Taxon classificationFungiHypocrealesNectriaceae

Sandoval-Denis, Crous & W.J. Swart sp. nov.

MB825104

Fig. 7

##### Diagnosis.

Different from most species in FSAMSC by its slender sporodochial conidia with tapered and somewhat rounded apex; its smooth- to tuberculate, often pigmented chlamydospores and the formation of large mycelial tufts on OA.

##### Type.

South Africa, Kruger National Park, Skukuza, Granite Supersite, 25°06'45.5"S, 31°34'35.0"E, from rhizosphere soil of *Sida cordifolia*, 23 Mar 2015, W.J. Swart, holotype CBS H-23497, dried culture on SNA, culture ex-holotype CBS 144211 = CPC 30923.

##### Description.

Colonies on PDA growing in the dark with an average radial growth rate of 8.5–9.3 mm/d, reaching 34–37 mm diam. in 7 d at 24 °C, filling an entire 9 cm Petri dish in 7 d at 27–33 °C. Minimum temperature for growth 12 °C, maximum 36 °C, optimal 27–30 °C. Colony surface at first white, turning coral to dark vinaceous with white periphery and abundant yellow hyphae at the centre; flat, velvety to woolly, with abundant aerial mycelium and erect hyphal strings reaching several mm tall; colony margins regular and filiform. Reverse with yellow, coral or dark vinaceous patches, coral diffusible pigments strongly produced between 15–30 °C, turning scarlet to orange at 33–36 °C. Colonies on CMA and OA incubated at 24 °C in the dark occupying an entire 9 cm Petri dish in 7 d. Colony surface coral, rust to chestnut coloured in irregular patches, flat, felty to woolly, aerial mycelium scarce on CMA, mostly as radially dispersed white patches, on OA aerial mycelium abundant, especially on the periphery of the colony, forming dense, pustule-like, white mycelial tufts, formed by abundant intermingled hyphae and chlamydospores, 1–1.5 cm tall, with flesh to coral coloured stipes; margins on CMA and OA regular. Reverse pale luteous with red to coral periphery. Sporulation abundant from conidiophores formed on the aerial mycelium, at the agar level and from sporodochia. *Conidiophores* on the aerial mycelium straight or flexuous, septate, smooth- and thin-walled, up to 150 μm tall, sometimes emerging from irregular, swollen, pigmented and rough-walled cells on the hyphae; simple or sparingly and irregularly branched, branches bearing terminal, rarely lateral monophialides or reduced to conidiogenous cells borne laterally on hyphae; *phialides* on the aerial conidiophores short ampulliform, subulate to subcylindrical, smooth- and thin-walled, (7–)9–14(–15) μm long, (3–)4–5 μm at the widest point, without periclinal thickening and with a minute, inconspicuous collarette; *conidia* formed on aerial conidiophores of two types: a) hyaline, obovoid, ellipsoidal to clavate, smooth- and thin-walled, 0–1-septate, 2–14 × 2–4 μm; b) lunate to short falcate with a pointed apex and a somewhat flattened base, smooth- and thin-walled, 3–5-septate. Three-septate conidia: (16–)18–27(–29) × 5–6 μm; four-septate conidia: 21–24(–25) × 5–6 μm; five-septate conidia: (25–)27–33 × 5–6 μm. *Sporodochia* cream to orange coloured, formed abundantly on the surface of carnation leaves and rarely on the agar surface, at first very small and sparse later becoming aggregated. *Conidiophores* in sporodochia 22–31 μm tall, irregularly branched, bearing clusters of 3–6 monophialides; *sporodochial phialides* doliiform to ampulliform, (5–)9–14(–18) × (3–)4–5 μm, smooth- and thin-walled, with periclinal thickening and a short apical collarette. *Sporodochial conidia* falcate, wedge-shaped, tapering towards both ends, markedly curved and robust; apical cell longer than the adjacent cell, pointed; basal cell distinctly notched, sometimes somewhat extended (1–)3–5(–6)-septate, hyaline, smooth- and thick-walled. One-septate conidia: 19 × 4 μm; three-septate conidia: 20–27(–28) × 5–7 μm; four-septate conidia: (29–)30–32 × 5–7 μm; five-septate conidia: (26–)29–41(–53) × 4–5(–6) μm; six-septate conidia: 36 × 7 μm; overall (19–)25.9–40(–53) × (3.5–)4–6(–7) μm. *Chlamydospores* abundant, hyaline or pigmented, smooth- to rough-walled or tuberculate, 7–8 μm diam., terminal or intercalary, solitary, in chains or in clusters.

**Figure 7.**
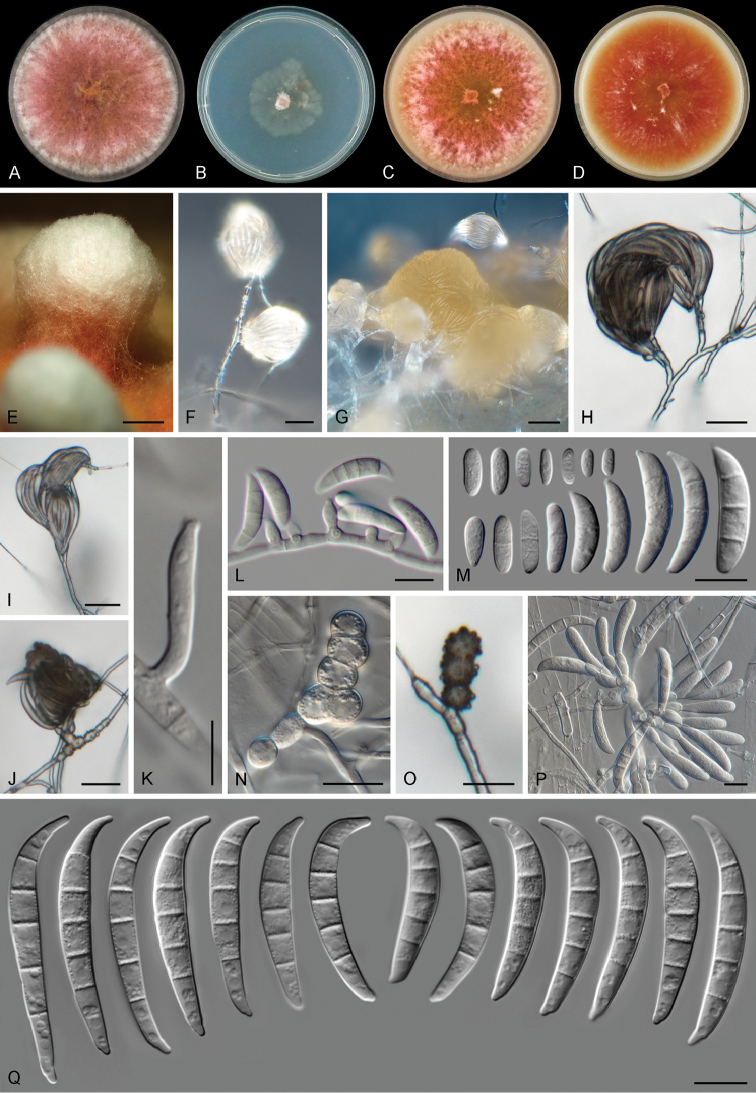
*Fusarium transvaalense* sp. nov. **A–D** Colonies on PDA, SNA, OA and CMA, respectively, after 7 d at 24 °C in the dark **E** Pustule-like growth on OA **F, G** Sporodochia formed on the surface of carnation leaves **H–L** Aerial conidiophores phialides and conidia **M** Aerial conidia **N, O** Chlamydospores **P** Sporodochial conidiophores and phialides **Q** Sporodochial conidia. Scale bars: 2 mm (**E**); 20 μm (**F–J**); 5 μm (**K**); 10 μm **(L–Q**).

##### Distribution.

Australia and South Africa

##### Etymology.

After Transvaal, the name of a former colony and Republic located between the Limpopo and Vaal rivers, currently a province of South Africa and where this species was found. From Latin *trans* meaning “on the other side of” and Vaal a South African river.

##### Additional isolates examined.

South Africa, Kruger National Park, Skukuza, Granite Supersite, 25°06'48.6"S, 31°34'36.5"E, from rhizosphere soil of *Melhania acuminata*, 23 Mar 2015, W.J. Swart, CBS 144224 = CPC 30928, CBS 144212 = CPC 30929); 25°06'45.6"S, 31°34'37.7"E, CBS 144496 = CPC 33750, CBS 144213 = CPC 33751; 25°06'48.8"S, 031°34'36.6"E, from rhizosphere soil of *Sida cordifolia*, 23 Mar 2015, W.J. Swart, CBS 144214 = CPC 30946; 25°06'45.7"S, 31°34'35.1"E, CBS 144215 = CPC 33723; 25°06'45.5"S, 31°34'35.0"E, CBS 144216 = CPC 30918, CBS 144217 = CPC 30919, CBS 144218 = CPC 30922, , CBS 144219 = CPC 30926, CBS 144220 = CPC 30927); 25°06'51.4"S, 31°34'37.5"E, from rhizosphere soil of *Kyphocarpa angustifolia*, 23 Mar 2015, W.J. Swart, CBS 144221 = CPC 33740; 25°06'51.8"S, 31°34'38.1"E, CBS 144222 = CPC 30939, CBS 144223 = CPC 30941.

##### Notes.

*Fusarium transvaalense* exhibits a sporodochial conidial morphology typical of members of FSAMSC with marked dorsiventral curvature and tapered ends. Several species in FSAMSC form comparable conidia in culture i.e. *F. crookwellense* L.W. Burgess, P.E. Nelson & Toussoun, *F. sambucinum*, *F. sporotrichioides* Sherb., *F. venenatum* Nirenberg and *F. culmorum* (Wm.G. Sm.) Sacc. However, with the exception of *F. sporotrichioides*, the conidia of most species above-mentioned, differ by being more robust and often more pointed apically. *Fusarium transvaalense* differs from *F. sporotrichioides* by the absence of pyriform aerial conidia.

Two strains NRRL 13829 and NRRL 31008, previously identified as *F. brachygibbosum* Padwick showed different degrees of genetic similitude with the new species. While NRRL 31008 clustered within *F. transvaalense*, NRRL 13829 formed a clearly delimited sister linage. Morphologically, *F. transvaalense* exhibits significant differences allowing its separation from *F. brachygibbosum*. Both species produce sporodochial conidia with similar septation and sizes; however, *F. brachygibbosum* commonly exhibits a bulge in the middle portion of the conidia (Padwick 1945), a feature not present in *F. transvaalense*. In addition, the latter species produces comparatively larger sporodochial conidia, when elements with the same degree of septation are compared; its chlamydospores are smaller, smooth-walled to markedly tuberculate and pigmented (7–8 μm vs. 10.7–15.3 μm, smooth-walled and hyaline in *F. brachygibbosum*) and has a distinctive colonial growth on OA, forming large, pustule-like hyphal tufts, a feature not reported for *F. brachygibbosum* (Padwick 1945).

## Discussion

In this study, three new *Fusarium* spp. were introduced, isolated from rhizosphere soils of three native African shrubs in a protected savannah ecosystem deep inside the Kruger National Park, South Africa.

Some remarkable differences were noted regarding the distribution of the novel fungal species and their respective hosts on this particular site. For instance, *F. transvaalense*, which exhibited the greatest relative abundance, was found in high quantities from the rhizospheres of the three hosts sampled, showing a considerable genetic diversity. Interestingly, this species was only on the top of the catena, even when two of its hosts, *K. angustifolia* and *S. cordifolia*, were found and sampled either at the top and bottom sites. Similarly, *F. fredkrugeri* was recovered only from soils under *M. acuminata*, a host species which occurred only at the top location. In contrast, *F. convolutans* was found in the rhizosphere of *K. angustifolia*, occurring only at the bottom of the catena, while none of the three fungal species was found associated with *S. cordifolia* at the bottom of the site. Nevertheless, not being an objective of this work, it was not possible to categorically assign these new species to specific hosts or locations. Likely, these fungi could be in low abundance and thus not detectable using the current methods. However, plant species composition varies considerably through a catena ecosystem, in relation to the different soil characteristics, pH gradient and water availability, which also greatly influence microbial and animal biodiversity (Lareen et al. 2016; Mohammadi et al. 2017). However, the full patterns of variation between locations on this particular catena still need to be systematically assessed and compared. As evidenced here, certain differences do exist between the soils at the upper and bottom locations of the Stevenson-Hamilton supersite, which might explain the fungal diversity variation observed here. The cation exchange capacity (CEC; capacity of a soil to hold exchangeable cations) varies considerably between sampling sites, basically depending on the proportion of sand versus clay content of each soil type (Ketterings et al. 2007; Van Zijl and Le Roux 2014). It is known that CEC greatly impacts the soil’s ability to retain essential nutrients and prevents soil acidification (Ketterings et al. 2007). Nutrient content also increased from the top to the bottom of the slope which is consistent with the increase in CEC. Nutrient poor soils are also a driver of biological diversity and most likely influenced fungal diversity in these particular locations (Havlicek and Mitchell 2014, Mapelli et al. 2017).

The three *Fusarium* species, described here, were not associated with any visible symptomatology on their hosts. However, they cannot be ruled out as pathogens since they were not assessed for pathogenicity against the sampled plants nor any other putative host species at the same locations. Likewise, it is unknown if these fungi exert any beneficial or deleterious effect on their ecosystems. These are important unsolved questions that need further evaluation. However, as shown by phylogenetic analyses, each of the three new species was in close genetic proximity with well-known plant pathogenic *Fusarium* spp. on their respective species complexes, which could suggest a potential pathogenic role. *Fusarium convolutans* clustered within the FBSC, together with three known plant pathogenic *Fusarium* spp. i.e. *F. buharicum*, a pathogen of *Hibiscus cannabinus* L. and *Gossypium* L.; *F. sublunatum*, known to affect banana and *Theobroma cacao* L. in Central America (Gerlach and Nirenberg 1982, Leslie and Summerell 2006) and a newly discovered although unnamed phylogenetic species causing wilt, crown and root rot of *Hibiscus moscheutos* L. (Lupien et al. 2017). *Fusarium transvaalense* belonged to the FSAMSC, a genetically diverse group common in temperate and subtropical zones (Leslie and Summerell 2006). *Fusarium sambucinum*, the conserved type species of the genus (Gams et al. 1997) being an aggressive plant pathogen and one of the most important agents of potato dry rot (Peters et al. 2008); while the latter species and several others in the complex have been reported causing disease on diverse crops, including many cereals and fruits (Leslie and Summerell 2006).

*Fusarium fredkrugeri* is here recognised and formally proposed as a new species. Although the clade representing this taxon had already been identified as a distinct unnamed phylogenetic species by O’Donnell et al. (2000), it had not been given a formal description pending the collection of additional isolates. Two other African isolates previously determined to belong to this clade i.e. CBS 144210 from *Striga hermonthica* (Del.) Benth. in Madagascar and NRRL 26152 from an unknown substrate in Niger, were incorporated into the analyses, although the latter strain is not viable anymore (NRRL, pers. comm.), thus not available for morphological assessment. Strain CBS 144210, however, is known as a pathogen of the ‘purple witchweed’, a parasite plant common to sub-Saharan Africa and known to devastate *Sorghum bicolor* (L.) Moench and *Oryza sativa* L. plantations (O’Donnell et al. 2000; Yoshida et al. 2010). As previously demonstrated by O’Donnell et al. (2000), our phylogenetic results showed that the clade comprising *F. fredkrugeri* and its sister species *F. dlaminii* does not cluster within the main African core of species in the FFSC. Thus, despite the African origin of our isolates, the predicted biogeographic patterns did not match the observed phylogeny. It has been hypothesised that this should not be the result of genetic markers tracing different phylogenies, but the consequence of losing the phylogenetic signal due to saturated sites and introns (O’Donnell et al. 2000). However, the inclusion in our analysis of additional, highly informative and slowly evolving loci such as *RPB1* and *RPB2* yielded similar results, which points out the need to re-evaluate the phylogeographic arrangement of this important species complex including the vast new data generated during the last 20 years that challenges the established assumptions (Kvas et al. 2009; Walsh et al. 2010; O’Donnell et al. 2013; Laurence et al. 2015). Nevertheless, although rather unlikely, alternative factors such as anthropogenic dispersion of *F. fredkrugeri*, its host or additional invasive alternative hosts, cannot be rejected as an explanation for the discordance between biogeography and phylogenetic results. However, these scenarios are difficult to imagine given the characteristics of the sampled site, not being an agroecosystem but a protected, isolated zone, with minimal human intervention (Smit et al. 2013).

This study is a new example of how easily new *Fusarium* spp. can be found when mycological studies are directed to neglected natural ecosystems of minimal anthropogenic disturbance (Phan et al. 2004; Leslie and Summerell 2011; Summerell et al. 2011; Burgess 2014, Laurence et al. 2015). Although irrelevant for some researchers, finding and properly describing new species, regardless of whether they have little or no pathogenic or mycotoxigenic potential, is of utmost importance to improve our understanding on the diversity, biogeographic and phylogeographic patterns of such a complex and heterogeneous genus as *Fusarium*. In addition, this study remarks on the significance and need to further stimulate the exploration of conserved, non-manipulated natural environments (supersites) and their potential impact on biodiversity research on the fungal kingdom.

## Supplementary Material

XML Treatment for Fusarium convolutans

XML Treatment for Fusarium fredkrugeri

XML Treatment for Fusarium transvaalense
